# Chronic Recurrent Multifocal Osteomyelitis: A Comprehensive Literature Review

**DOI:** 10.7759/cureus.43118

**Published:** 2023-08-08

**Authors:** Mariam Hassan, Heabah Assi, Maha Hassan, Jared J Bies, Swathi Prakash, Ali Hassan, Sara Alhariri, Fatma Dihowm

**Affiliations:** 1 Internal Medicine, Texas Tech University Health Sciences Center Paul L. Foster School of Medicine, El Paso, USA; 2 Biomedical Sciences, Gulf Medical University, Ajman, ARE; 3 Emergency Medicine, Mercy Health St. Elizabeth Boardman Hospital, Boardman, USA

**Keywords:** systemic inflammation, bone pain, aseptic inflammatory bone disorder, noninfectious osteomyelitis, chronic recurrent multifocal osteomyelitis

## Abstract

Chronic recurrent multifocal osteomyelitis (CRMO) is a non-infectious, inflammatory disorder of the bones. CRMO typically affects children, with a predisposition to females. Bone-related pain is often felt in the metaphysis of long bones, particularly of the lower extremities, but it can also target other sites at varied time intervals. Patients are likely to complain of tenderness and swelling that may cause considerable disability and adversely impact quality of life. There are three main pathophysiological mechanisms that have been hypothesized to drive CRMO including imbalanced cytokine expression, increased inflammasome activation, and enhanced osteoclast differentiation. Therapies have been based on targeting and suppressing these key players in CRMO patients. The first step in management involves pain control. Non-steroidal anti-inflammatory drugs should provide initial relief, albeit temporarily. It is imperative to initiate immunosuppressive medication that will help limit bone involvement and thereby prevent the development of fractures or leg-length discrepancies, for example. The purpose of this literature review is to study the pathophysiology of CRMO and carefully dissect the agents that have been previously employed in the management of CRMO patients. This could allow for the purposeful formulation of individualized care plans and improving the overall well-being of patients. The authors included a multitude of PubMed-indexed articles published from 2000 onwards in this review.

## Introduction and background

Chronic recurrent multifocal osteomyelitis (CRMO) was first described in 1972 by Giedion as “an unusual form of multifocal bone lesions with subacute and chronic symmetrical osteomyelitis” [1]. It is the most severe presentation on the spectrum of chronic nonbacterial osteomyelitis (CNO) [2,3]. CRMO is an insidious, sterile, autoinflammatory disorder that affects the bones in children and adolescents with a waxing and waning character, but is typically worse at night [1,2,4].

Autoinflammatory disorders such as CRMO are characterized by an activation of the innate immune system in the absence of high-titer autoantibodies, pathogens, or antigen-specific T-cells [1,2]. The bone lesions mimic infectious osteomyelitis histologically and radiographically, but manifestations improve with the use of anti-inflammatory medications, as opposed to antibiotics [3,5]. Patients will complain of pain that ranges on a wide clinical spectrum, from mild, time-limited, and monofocal bone discomfort to severe, recurrent, multifocal inflammation and disabling pain [2]. A consistent presenting feature of CRMO is the gradual worsening of pain, swelling, and tenderness localized to areas of affected bones [1]. A thorough literature review substantiates the affliction of long bones, namely the clavicle, metaphyses, and epiphyses of the femur, tibia, or humerus [1]. Patients can also experience pain in the pelvic bones, vertebral column, or the shoulder girdle/clavicle [2].

Despite the incidence being reported as low as four per one million children, it has been on a steady rise in recent years as the medical community familiarizes itself with this relatively obscure condition [6]. The peak onset of the disease is 7-12 years of age, and it often affects young females more than males [2,6]. Although CRMO lacks predilection for geographical regions, White Caucasians are the most frequently reported race in literature [2,6]. However, global epidemiologic studies are sparse and only include small case series and regional cohorts, which can affect our understanding of epidemiology.

A genetic predisposition has been suggested by familial clustering and a strong association with inflammatory disorders of the skin and intestinal tract in affected individuals and among close relatives [7]. The families of patients with CRMO are noted to harbor inflammatory conditions such as Crohn’s disease, ulcerative colitis, celiac disease, and ankylosing spondylitis [2-4,7]. Skin conditions include palmoplantar pustulosis, psoriasis vulgaris, severe acne, generalized pustulosis, and Sweet syndrome [2-4,7,8].

## Review

Pathophysiology

The molecular pathophysiology underlying CRMO is not completely understood. However, a general consensus of the literature highlights the pivotal role of cytokine dysregulation in facilitating the “sporadic”, or non-familial, form of CNO [2].

The temporal and spatial regulation of cytokine expression is not only essential in controlling inflammation but also osteoprogenitor cell differentiation. The loss of homeostasis between pro- and anti-inflammatory cytokines can have powerful impacts on driving bone remodeling in the direction of resorption [9]. This process is largely dependent on the osteoprotegerin (OPG)/receptor activator of the nuclear factor-κB ligand (RANKL) axis. The receptor activator of nuclear factor-kB (RANK) is expressed by osteoclast precursors, and it binds the RANK ligand (RANKL) on osteoblast surface membranes. This binding initiates the differentiation of the osteoclast progenitor into a mature, resorbing osteoclast [9]. Meanwhile, the protein OPG produced by osteoblasts inhibits this process by binding to RANKL, thereby preventing the activation of RANKL [10].

Various studies have suggested that pro-inflammatory cytokines, mainly tumor necrosis factor-alpha (TNFα) and interleukin (IL)-1β, promote this osteoclastogenesis via the upregulation of RANKL in osteoblasts and bone marrow macrophages (BMM) [11-13]. Another study suggests that TNFα can disrupt the differentiation of osteoblast, skewing the balance in bone remodeling towards resorption [14]. On the other hand, the anti-inflammatory cytokine IL-10 has been shown to downregulate RANKL and upregulate OPG expression, thereby promoting bone formation [15]. In the context of CRMO, the imbalance between pro-inflammatory cytokines (IL-1β, IL-6, TNFα) and anti-inflammatory cytokines (IL-10, IL-19) have been linked with reduced activation of the mitogen-activated protein kinases (MAPKs)/extracellular signal-regulated kinase (ERK) signaling pathway.

Previously, it was demonstrated that monocytes derived from CRMO patients had impaired IL-10 expression in response to toll-like receptor 4 (TLR-4) stimulation by lipopolysaccharide (LPS) [16]. The attenuated MAPKs/ERK signaling resulted in decreased nuclear shuttling of the transcription factor signaling protein 1 (SP-1) and, by extension, diminished binding of SP-1 to the IL-10 promoter region. Additionally, the alteration in ERK signaling led to epigenetic modification at the IL-10 promoter; the reduced histone H3 serine 10 (H3S10) phosphorylation further hindered IL-10 expression. It was proposed that the same mechanisms may account for the lower IL-19 expression as well [17].

Downregulated IL-10 may contribute to osteoclastogenesis by enabling increased inflammasome activation, particularly, the NLR family pyrin domain containing 3 (NLRP3) inflammasome. This increase has been depicted through the elevated mRNA levels of proteins encoding the inflammasome components apoptosis-associated speck-like protein containing a caspase recruitment domain or CARD (ASC), NLRP3, and caspase-1 [18]. This assembly of proteins plays a role in the activation of IL-1β, a pro-inflammatory cytokine whose expression is also commonly elevated in peripheral blood mononuclear cells (PBMCs) isolated from CRMO patients [18]. Consequently, an inverse relationship between anti- and pro-inflammatory cytokine levels manifests. In the monocytes of CRMO patients, the decrease in inflammasome activation upon co-culture with recombinant IL-10 further substantiates the suggested immunomodulatory function of IL-10 on inflammasomes [18]. The same was also observed in IL-10-deficient mice and was linked with inflammatory bone resorption [19].

Apart from the aforementioned molecular signaling regulation of IL-10 expression, genetic variants of the IL-10 promoter region may also serve in predetermining transcription levels. Three main promoter haplotype blocks (GCC, ACC, and ATA) have been identified and associated with the ability of the IL-10 promoter to recruit the SP-1 transcription factor (high to low). Surprisingly, the majority of CRMO patients in the cohort exhibited the haplotype blocks which encoded for “high” IL-10 expression (GCC) [2]. While they were not able to scientifically confirm the reasoning behind this, the authors hypothesized that CRMO patients with “low” expression IL-10 promoter haplotype blocks (ACC and ATA) may have developed and been diagnosed with different and more severe autoinflammatory conditions.

The “monogenic” or familial autoinflammatory disorders of CNO and their related mutations are also associated with IL-1β dysregulation. For instance, the *LPIN2* gene mutation in Majeed syndrome, characterized by CRMO, results in defective P2X47 receptors on macrophages, leading to upregulation of NLRP3 inflammasomes and heightened activation of IL-1β [4]. Similarly, the proline-serine-threonine phosphatase interacting protein 1 (PSTPIP) mutation in pyogenic arthritis, pyoderma gangrenosum, and acne (PAPA) syndrome causes greater activation of the pyrin inflammasome and elevated IL-1β levels [20]. Deficiency of IL-1 receptor antagonist (DIRA) syndrome also manifests with elevated IL-1β levels; however, this is due to mutations in the *IL1RN* gene [4]. It should be noted that most CRMO patients do not present with monogenic disease [6]. Nonetheless, efforts have been dedicated to investigating possible genetic predispositions in CRMO cohorts.

Two CNO/CRMO susceptibility genes have recently been identified using whole exome sequencing. Mutations in the *FBLIM1* gene were identified in two unrelated South Asian CRMO patients and were implicated in promoting osteoclast differentiation and bone resorption by precluding the regulation of RANKL activation [21]. Both patients were reported to have the haplotype blocks associated with “low” IL-10 expression. Considering the fact that FBLIM1 expression is mediated by STAT3 signaling, which is dependent on IL-10 activation, it has been suggested that their low IL-10 expression may possibly contribute to the filamin binding LIM protein 1 (FBLIM1) loss of function [21]. Another two mutations in the Gardner-Rasheed feline sarcoma viral (v-fgr, or* FGR*) gene, encoding for an Scr kinase family member, have also exhibited supporting roles in the pathogenesis of aseptic bone inflammation in two CRMO patients as well as mouse models [22]. However, this contribution was made through mast cells and neutrophils, where the *FGR* gene is predominantly expressed and was illustrated to be done independently of the NLRP3 inflammasome.

Overall, the main three pathophysiological mechanisms that have been hypothesized to drive CRMO include imbalanced cytokine expression, increased inflammasome activation, and enhanced osteoclast differentiation [23]. Thus, therapies have been based on targeting and suppressing these key players in CRMO patients.

Diagnosis

The clinical suspicion for CRMO will be based on age, symptoms, and/or family history that is suggestive of CRMO or human leukocyte antigen B27 (HLA-B27) positive conditions [4,5]. The ultimate diagnosis, however, is made by the exclusion of other diseases and subsequent bone biopsy [2,4,6]. Differential diagnoses include, but are not limited to, non-specific musculoskeletal disorders, infectious causes (bacterial osteomyelitis, mycobacteria, viral and fungal etiologies), malignancy (Ewing’s sarcoma, Langerhans’ cell histiocytosis, metastatic lesions), benign tumors (osteoid osteoma, bone cysts, fibrosis), other autoinflammatory disorders (juvenile idiopathic arthritis, PAPA (pyogenic arthritis, pyoderma gangrenosum, and acne), DIRA (deficiency of interleukin-1 receptor antagonist), Majeed syndrome), osteonecrosis, osteopetrosis, and more [2,4,6].

The musculoskeletal examination may be normal except for tenderness and possible swelling of the affected bone site [1,5]. A laboratory workup could reveal findings suggestive of anemia of chronic disease with normal or mild elevation in inflammatory markers, such as erythrocyte sedimentation rate (ESR) and C-reactive protein (CRP) [2,4,5]. It is imperative to note that a subset of patients will not exhibit any serological proof of systemic inflammation [2]. TNFα levels may also be elevated [5].

Radiography is obtained to rule out fractures and signs of infection [2]. Early radiographic images can be negative for any abnormality [2,4]. Alternatively, the inflammatory bone lesions that are detected in plain X-rays will present as radiolucent, osteolytic, or sclerotic lesions, depending on the disease stage [2,4]. The Bristol Royal Hospital for Children reviewed data for children (aged <18 years) diagnosed with CRMO between January 2005 and December 2012 [1]. A plain radiograph of a major symptomatic site was obtained in 36 out of 41 patients. Of these 36 patients, 50% demonstrated lytic lesions in the plain radiographs, 53% showed areas of sclerotic bone, and 33% revealed a periosteal reaction [1].

Magnetic resonance imaging (MRI) is the most sensitive diagnostic tool to identify ongoing inflammation in particular bone segments [2,5]. MRI is especially sensitive during the early stages of inflammation, and bone edema will be apparent even before erosions and/or sclerosis are appreciated [2,4]. In the reparative phase, areas of metaphyseal and epiphyseal destruction heal via sclerosis. This cycle of destruction and reparation results in progressive hyperostosis and sclerosis of the metaphysis and diaphysis [6].

A bone biopsy is pursued if suspecting or attempting to rule out infection, neoplasia, or other systemic disease [2,4]. The bone biopsy will result in lesions with histologic features that mimic osteomyelitis but with negative cultures [1]. Histopathological evaluation of early lesions predominantly consists of neutrophils, clusters of lymphocytes, and occasional eosinophils surrounding small abscesses [4,5]. In chronic lesions, lymphocytes with plasma cells, histiocytes, and some polymorphonuclear cells are typically visualized [4,5]. Necrotic bone fragments, fibrosis, and increased occurrence of osteoblasts with dilated blood vessels are seen in previously established lesions [4].

Recommendations for management

There are several treatment options available for the management of patients with CRMO and that include, but are not limited to, non-steroidal anti-inflammatory drugs (NSAIDs), corticosteroids, bisphosphonates, anti-TNF agents, or disease-modifying anti-rheumatic drugs (DMARDs), such as methotrexate or sulfasalazine.

NSAIDs are employed as first-line therapy in patients without involvement of the vertebral column. These analgesics are effective in a large subset of patients within the first o ne to two years of treatment, providing quick symptomatic relief and helping control bone inflammation [2,6]. Moreover, NSAIDs are thought to alter disease course due to prostaglandins' involvement in osteoclast activation and bone remodeling. Regardless, more than 50% of patients experience a flare after two years of treatment initiation and require an alternate treatment [2].

Corticosteroids are thought to function in a similar manner to NSAIDs, but through inhibition of phospholipase A1 to reduce the production of prostaglandins [6]. Furthermore, corticosteroids have a negative effect on the expression of proinflammatory cytokines regulated by transcription factor NFκB, including IL-1, IL-6, and TNFα [6]. In clinical practice, short oral courses of 2 mg/kg/day prednisone equivalent can be administered over 5-10 days [5,6]. Alternatively, an even lower dose (0.1-0.2 mg/kg/day) can be used as a bridging treatment until other therapies for CRMO are introduced. A thorough review of virtually accessible literature highlights favorable effects in a majority of patients, but long-term use of corticosteroids will demonstrate considerable side effects [5,6].

Bisphosphonates, mainly pamidronate, have shown increasing efficacy when NSAIDs have failed to control symptoms, reducing pain and improving function [4,5,24]. Bisphosphonates are known to inhibit osteoclasts and may reduce lesion expansion via an unknown mechanism of action [8]. MRI evidence showed a reduction in active lesions and has contributed to the evidence of benefit [8]. One study showed six months as the mean time of MRI resolution of bone inflammation, with a range of 2-12 months [4].

Anti-TNF agents, such as etanercept or anakinra, are another type of treatment for CRMO [24]. TNFα is expressed in increased levels from monocytes in CRMO patients and has shown some benefit for patients [25,26]. However, given the off-label use and the high cost of TNF inhibitors along with safety concerns, it is recommended to reserve use for complicated cases [5,25].

DMARDs, such as methotrexate, sulfasalazine, and leflunomide, have also become increasingly used as a treatment in the past few years but information regarding their benefit is sparse [25]. IL-1 blocking strategies seem to be a promising therapy due to increased NLRP3 inflammasome assembly, but studies have shown conflicting results [6]. There has been increased use of these drugs in the past decade as part of a potential combination treatment in patients with CRMO [25].

Duration and discontinuation of treatment remain unknown due to the absence of published evidence and the chronic nature of the disease [4,6].

Discussion

CRMO, also referred to as CNO, is a non-infectious, autoinflammatory disease process. The time to diagnosis is often delayed and mistreated due to its rarity and being a diagnosis of exclusion. This condition develops more commonly in the adolescent range and in females. One study reported 41 patients diagnosed with CRMO within an eight-year period, with first symptom onset occurring at a median of nine years (range 1-13), the median age at diagnosis being 11 years (range 1-17), and time to diagnosis had a median of 15 months (range 0-92) [1]. This delay in diagnosis and appropriate treatment presents a major challenge for CRMO patients by leading to a prolonged course of antibiotics with inpatient care, unnecessary radiation exposure from multiple imaging studies, and repeated surgeries including bone biopsies.

Studies have also shown that NSAIDs are considered the first line for patients with non-vertebral CRMO [2,6]. This can be effective for patients, but in the event of a flare-up, it may warrant an escalation of analgesia that includes corticosteroids, biologics, or DMARDs. Corticosteroids minimize the autoinflammatory process that comprises the pathogenesis of CRMO. On the other hand, corticosteroids are not ideal for long treatment courses given their side effects profile [5,6]. The commencement of biologics, such as etanercept or anakinra, has benefits in the clinical response in patients with CRMO, but these medications are very expensive [5,25,26]. Additionally, starting biologics requires testing for tuberculosis prior to starting treatment, and routine follow-up to monitor clinical response and the development of adverse effects, as well. DMARDs are another class of medications that have been trialed for patients with CRMO, but their effectiveness remains unclear [25]. Both biologics and DMARDs predispose patients to an increased risk for infections given their immunosuppressive effects. There is a lack of prospective double-blind, randomized, clinical trials analyzing the clinical effect and side effects of biologics and DMARDs in patients with CRMO, and this is imperative for devising a standardized, international blueprint that will better guide clinicians through treatment.

Despite repeated treatment courses in patients with CRMO, clinical symptoms may disappear for a short period of time before returning, while in other cases, symptoms may permanently disappear. For others with controlled CRMO, chronic pain in the form of amplified musculoskeletal pain not responsive to medications can manifest and require referral to a pain management clinic. This manifestation of uncontrolled pain in patients with CRMO highlights the psychosocial aspect of this rare diagnosis. Initially, treatment may be initiated on an experimental basis, and monitoring response to any single agent could take weeks. This can be emotionally exhausting for patients and their families as they wait for a final diagnosis.

Once the diagnosis has been established and pain control is addressed, patients require routine monitoring of disease activity through repeat blood work, and repeat MRIs through childhood and early adulthood are done to assess the resolution of active bone lesions and/or detect new lesions. These patients have a close follow-up with a rheumatologist to monitor clinical status. For some patients, activity limitations are necessary to prevent serious injury and further skeletal damage. Patients with CRMO spine involvement are at increased risk for serious neurological injury if vertebral fractures were to occur. CRMO spine involvement can be complicated by vertebral fractures, kyphosis, and leg length discrepancy. Therefore, patients with CRMO must discuss activity restrictions with their provider to negate risks that could lead to devastating neurological sequelae.

## Conclusions

The hallmark of CRMO, an autoinflammatory disorder, is sterile bone inflammation that is recurrent and may affect various bones at once. CRMO is vastly underdiagnosed due to a lack of awareness among medical professionals and the general population, the absence of validated diagnostic criteria or disease biomarkers, and the difficulty of collecting data in countries without national registries. The management involves trialing various NSAIDs as well as disease-modifying agents to slow progression and prevent bone destruction from occurring. Several pharmaceutical options are available in the rheumatological field that can be beneficial; however, all are accompanied by adverse drug reactions that will require routine monitoring. Patients' physical activity is usually limited secondary to pain and leading to lower quality of life. Thus, the purpose of this literature review is to raise awareness of a condition that mandates timely diagnosis, explore attempted management strategies that can be employed in future encounters, and discuss the important psychosocial implications of CRMO on children or adolescents.
